# The Association between Sexually Transmitted Infections, Length of Service and Other Demographic Factors in the U.S. Military

**DOI:** 10.1371/journal.pone.0167892

**Published:** 2016-12-09

**Authors:** Robert Deiss, Richard J. Bower, Edgie Co, Octavio Mesner, Jose L. Sanchez, Jennifer Masel, Anuradha Ganesan, Grace E. Macalino, Brian K. Agan

**Affiliations:** 1 Infectious Disease Clinical Research Program, Department of Preventive Medicine and Biostatistics, Uniformed Services University of the Health Sciences, Bethesda, Maryland, United States of America; 2 The Henry M. Jackson Foundation for the Advancement of Military Medicine, Bethesda, Maryland, United States of America; 3 Naval Medical Center San Diego, San Diego, California, United States of America; 4 William Beaumont Army Medical Center, El Paso, Texas, United States of America; 5 Armed Forces Health Surveillance Branch, Public Health Division, Defense Health Agency, Silver Spring, Maryland, United States of America; 6 Walter Reed National Military Medical Center, Bethesda, Maryland, United States of America; Asociacion Civil Impacta Salud y Educacion, PERU

## Abstract

**Background:**

Numerous studies have found higher rates of sexually transmitted infections (STIs) among military personnel than the general population, but the cumulative risk of acquiring STIs throughout an individual’s military career has not been described.

**Methods:**

Using ICD-9 diagnosis codes, we analyzed the medical records of 100,005 individuals from all service branches, divided in equal cohorts (n = 6,667) between 1997 and 2011. As women receive frequent STI screening compared to men, these groups were analyzed separately. Incidence rates were calculated for pathogen-specific STIs along with syndromic diagnoses. Descriptive statistics were used to characterize the individuals within each accession year cohort; repeat infections were censored.

**Results:**

The total sample included 29,010 females and 70,995 males. The STI incidence rates (per 100 person-years) for women and men, respectively, were as follows: chlamydia (3.5 and 0.7), gonorrhea (1.1 and 0.4), HIV (0.04 and 0.07) and syphilis (0.14 and 0.15). During the study period, 22% of women and 3.3% of men received a pathogen-specific STI diagnosis; inclusion of syndromic diagnoses increased STI prevalence to 41% and 5.5%, respectively. In multivariate analyses, factors associated with etiologic and syndromic STIs among women included African American race, younger age and fewer years of education. In the overall sample, increasing number of years of service was associated with an increased likelihood of an STI diagnosis (p<0.001 for trend).

**Conclusion:**

In this survey of military personnel, we found very high rates of STI acquisition throughout military service, especially among women, demonstrating that STI-related risk is significant and ongoing throughout military service. Lower STI incidence rates among men may represent under-diagnosis and demonstrate a need for enhancing male-directed screening and diagnostic interventions.

## Introduction

Sexually transmitted infections (STIs) have been a longstanding problem in the United States military. Prior to the discovery of antibiotics, these infections were a significant cause of morbidity and mortality, with a significant toll on operational readiness; in fact, before the invention of modern weaponry, venereal disease was more commonly lethal than combat duty [1]. During World War I, STIs were only second to influenza in disabling troops from performing their duties, with 7 million person days lost to STIs in the US army [2]. In recent decades, the influence of human immunodeficiency virus (HIV), increased numbers of deployed personnel and enhanced tools for STI screening and diagnosis have necessitated ongoing attention to the burden of STIs.

There are many reasons why STI diagnoses are prevalent among military personnel, including the population of healthy, sexually active, risk taking young adults. Demographic characteristics commonly associated with increased risk for STIs, including African-American race, younger age, education at the high school (as opposed to college) level and residence in endemic areas [3,4], are over-represented in the military compared to the general population. Thus, even at accession, military recruits show a high rate of STIs prior to starting their service [5]. In addition, military service may appeal to individuals prone to risk-seeking behavior, and high prevalence of sexual risk behavior is well-described [6,7]. Last, military service provides more opportunities for health screening, resulting in higher ascertainment of diagnoses; the disparity is particularly noteworthy among women in the military, who receive annual gonorrhea/chlamydia screening, and therefore have higher rates of STI diagnosis than men, who are only screened for HIV infection.

Despite frequent screening and surveillance, it is often difficult to determine the longitudinal impact of STIs on the military. Several studies have found high rates of reinfection among individuals with STIs [3,8], but the cumulative risk over an individual’s military career is less well-described. This paper takes a novel approach to this problem by selecting cohorts of service-members based on the year they joined the armed forces and evaluating the occurrence of STI diagnoses over the course of their service based on administrative records in the centralized military healthcare system. Our goal was to gain a better understanding of the prevalence of sexually transmitted infections in the DOD, to identify where more data are needed, and to potentially identify areas for intervention.

## Materials and Methods

We retrospectively studied data which were collected from the Defense Medical Surveillance System (DMSS) between 1997–2012. The DMSS is a centralized relational database which contains medical encounter data for the US armed forces, including clinical, laboratory and epidemiologic data [9]. We sampled 100,005 subjects in cohorts of 6,667 based on year of entry into military service; all cohorts had at least one year of follow-up. We oversampled females by a 2:1 ratio to balance the likely outcome counts: while females represent 14.5% of military personnel, they experience approximately twice the rate of STIs of males. We obtained demographic information, including age at accession, sex, race, home of record (zip code), marital status and military-specific data including date of accession/discharge, service affiliation, job code/rank and deployment status. Medical information included all STI diagnoses (at any anatomic site), hepatitis B virus (HBV) and human papilloma virus (HPV) immunization dates and exemptions, and the date of the first DoD seropositive test among HIV-infected individuals. Both etiologic and syndromic STI diagnoses were considered in our search (see definitions, Table 1).

**Table 1:** ICD-9 Diagnosis Codes examined in this study.

Condition	Diagnosis Code
Etiologic	
*Chlamydia trachomatis*	099.41, 099.5
*Neisseria gonorrhoeae*	098, V02.7
Herpes simplex virus (HSV-genital)	054.1,
Human papilloma virus (HPV-genital)	078.1, 079.4, 795.05, 795.09, 795.15, 795.19, 796.75, 796.79, V73.81
Syphilis	090, 091, 092, 093, 094, 095, 096, 097
*Lymphogranuloma venereum* (LGV)	991
Trichomoniasis	131
Hepatitis B virus (HBV)	0703, 0702
Human immunodeficiency virus (HIV)	V08, 042
Syndromic	
Cervicitis (unspecified agent, <45yo)	616.0
Vaginitis (<45yo)	616.1
Pelvic Inflammatory Disease (PID)	614
Orchitis/Epididymitis, Prostatitis (<35yo)	604, 601
Urethritis (<35yo)	099.4

### Data analysis

Descriptive statistics were used to characterize the accession year cohorts. Incident STI diagnoses were counted by each quarter of a calendar year. A bacterial STI was considered incident and only counted once in each quarter. Viral STIs were considered incident only at the first recorded diagnosis. If a syndromic diagnosis was found in the same quarter as an etiologic diagnosis explaining the syndrome (e.g. gonorrhea and urethritis), only the etiologic diagnosis was counted. Incidence rates and unadjusted risk ratios were calculated for each STI diagnosis, and a multivariate Poisson regression was performed to determine the relationship between number of STIs and years in the military. In addition, multivariate logistic regression was used to characterize factors independently associated with a history of diagnosed syndromic or pathogen-specific STIs.

All patient demographics and medical records were anonymous and de-identified prior to analysis. All relevant data are included within the manuscript. This study was approved by the Institutional Review Board of the Uniformed Services University of the Health Sciences.

## Results

The total sample size included 70,995 males (71%) and 21,005 females (29%), with baseline demographics shown in Table 2. Owing to the large number of subjects in the sample, all variables of interest demonstrated statistically significant differences in univariate analyses. STI rates were highest among women, African-Americans, enlisted personnel and Air Force/Army compared with other service branches (Table 3). STI rates were higher among individuals without a history of deployment, except for pathogen-specific diagnoses (Table 3), though the end-study prevalence of STIs was higher among individuals who had been deployed than individuals with a history of non-deployment (Table 2).

**Table 2:** Baseline characteristics of participants and lifetime prevalence of at least one sexually transmitted infection.

	Overall–women N (%)	Syndromic—women N (%)	Etiologic—women N (%)	Overall—men N (%)	Syndromic—men N (%)	Etiologic—men N (%)
**Median Age at entry (IQR)**^1^	19 (18–22)	19 (18–21)	19 (18–21)	19(18–21)	19 (18–21)	19 (18–21)
**Race-White**	17,876	4,823 (26.9)	2,791 (15.6)	51,994	1,266 (2.4)	1,248 (2.4)
**Race-Black**	6,813	3,254 (47.8)	1,844 (27.0)	9,874	247 (2.5)	789 (8.0)
**Race-Other**	2,789	780 (28.0)	526 (18.9)	5,567	108 (1.9)	144 (2.6)
**Race-Unknown**	1,532	597 (39.0)	324 (21.1)	3,560	130 (3.7)	143 (4.0)
**Rank- Enlisted**	11,193 (41.4)	8,526 (31.6)	5,237 (19.4)	3,753 (5.5)	1,654 (2.4)	2,247 (3.3)
**Officer**	606 (30.5)	443 (22.3)	248 (12.5)	160 (5.0)	86 (2.7)	77 (2.4)
**History of deployment**	5,251 (53.4)	4,026 (41.0)	2,709 (27.6)	2,465 (6.9)	1,091 (3.1)	1,481 (4.1)
**No history of deployment**	6,548 (34.1)	4,943 (25.8)	2,776 (14.5)	1,448 (4.1)	660 (1.9)	843 (2.4)
**Single**	10,123 (40.8)	8,038 (32.4)	4,838 (19.5)	3,509 (5.5)	1,506 (2.4)	2,150 (3.4)
**Married**	1,426 (40.5)	1,205 (34.2)	542 (15.4)	347 (5.5)	210 (3.3)	150 (2.4)
**Air Force**	3,233 (41.8)	2,480 (32.0)	1,463 (18.9)	780 (5.9)	407 (3.1)	403 (3.1)
**Army**	4,924 (42.3)	3,930 (33.8)	2,138 (18.4)	1,638 (6.0)	666 (2.4)	1,042 (3.8)
**Marines**	706 (30.9)	485 (21.3)	364 (16.0)	627 (4.5)	290 (2.1)	359 (2.6)
**Navy**	2,936 (39.9)	2,074 (28.2)	1,520 (20.7)	867 (5.2)	376 (2.3)	520 (3.1)
**Median years of service**^1^	4 (1.8–6.3)	5.3 (3.4,8.5)	5.7 (3.7,8.7)	4(2.4.6.9)	6.3(4,11)	6.2 (4–10)

^1^Interquartile range

**Table 3:** Incident rates (per 1,000 person-years) of sexually transmitted infections for women and men.

	Syndromic—women (95% CI)	Etiologic—women (95% CI)	Syndromic—men (95% CI)	Etiologic—men (95% CI)
**Median Age at entry (IQR)**^1^				
**18**	40.1 (39.2, 41.0)	15.7 (15.1, 16.3)	1.5 (1.4, 1.6)	2.2 (2.1, 2.4)
**19**	38.7 (37.3, 40.0)	16.1 (15.3, 17.0)	1.8 (1.6, 1.9)	2.3 (2.1, 2.5)
**20–21**	38.4 (37.1, 39.6)	14.1 (13.4, 14.9)	1.8 (1.6, 2.0)	2.2 (2.0, 2.3)
**22+**	34.4 (33.4, 35.4)	10.4 (9.9, 11.0)	1.7 (1.5, 1.8)	1.6 (1.4, 1.7)
**Race-White**	28.3 (27.7, 29.0)	11.7 (11.3, 12.1)	1.7 (1.6, 1.9)	1.5 (1.4, 1.5)
**Race-Black**	64.4 (63.0, 65.9)	20.3 (19.6, 21.1)	1.6 (1.4, 1.8)	5.3 (5.0, 5.6)
**Race-Other**	28.4 (26.9, 30.0)	13.7 (12.7, 14.9)	1.3 (1.1, 1.6)	1.7 (1.4, 1.9)
**Race-Unknown**	34.1 (32.2, 36.1)	10.9 (9.8, 12.0)	2.2 (1.9, 2.5)	2.0 (1.7, 2.3)
**Rank-Officer**	16.7 (15.5, 17.9)	6.1 (5.4, 6.9)	1.2 (1.0, 1.5)	1.0 (0.8, 1.2)
**Enlisted**	40.1 (39.6, 40.8)	14.9 (14.5, 15.2)	1.7 (1.6, 1.8)	2.1 (2.1, 2.2)
**History of deployment**	35.4 (34.7, 36.1)	13.3 (12.8, 13.7)	1.5 (1.5, 1.6)	2.4 (2.2, 2.5)
**No history of deployment**	40.9 (40.1, 41.7)	15.0 (14.5, 15.5)	1.9 (1.8, 2.1)	1.9 (1.8, 2.0)
**Single**	37.2 (36.6, 37.8)	14.3 (14.0, 14.7)	1.6 (1.5, 1.7)	2.1 (2.1, 2.2)
**Married**	44.1 (42.3, 45.9)	12.8 (11.9, 13.9)	2.4 (2.1, 2.7)	1.0 (0.8, 1.2)
**Air Force**	33.8 (32.9, 34.7)	11.3 (10.9, 11.9)	1.9 (1.7, 2.0)	1.6 (1.4, 1.7)
**Army**	48.5 (47.4, 49.5)	16.0 (15.3, 16.7)	1.7 (1.5, 1.8)	2.6 (2.4, 2.7)
**Marines**	25.2 (23.5, 27.0)	12.7 (11.5, 14.0)	1.7 (1.5, 1.8)	1.7 (1.6, 1.9)
**Navy**	31.9 (30.9, 32.9)	15.1 (14.4, 15.8)	1.5 (1.3, 1.6)	2.0 (1.8, 2.1)

^1^Interquartile range

**Fig 1:**
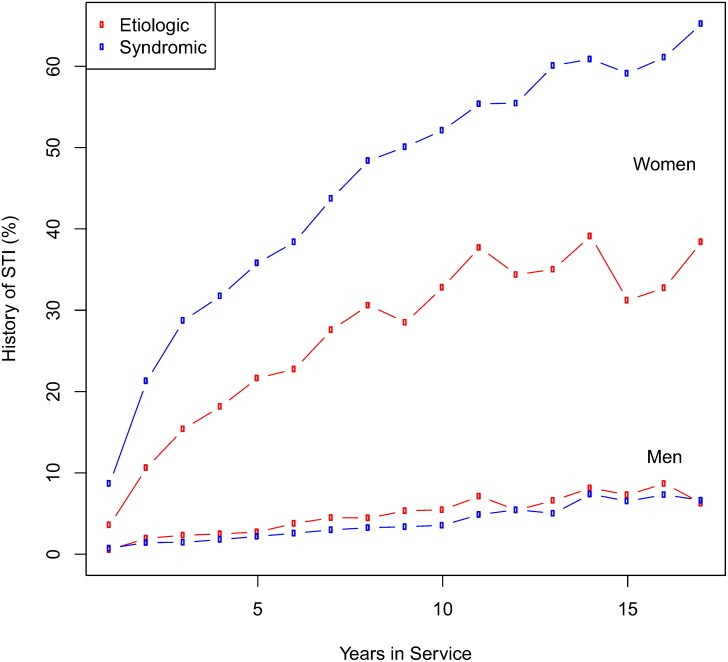
Percentage of individuals with a sexually transmitted infection by year of service.

The career prevalence of ever being diagnosed with an STI is depicted in Fig 1. For both men and women, increasing number of years of service was associated with an increased proportion experiencing an STI. After 15 years of service, 41% of women and 6% of men had experienced at least one STI. Among women, 33% were diagnosed with a syndromic STI, and 19% were diagnosed with a pathogen-specific STI. Among men, these rates were 3% and 2%, respectively. Over the course of the study, the proportion of syndromic diagnoses decreased from 1997–2014, while pathogen-specific diagnoses increased (data not shown).

The distribution of STIs among males and females is shown in Table 4. With the exception of HIV, pathogen-specific STI rates were higher among women than men. Among women, infection with human papilloma virus (HPV) was the most common pathogen-specific diagnosis, followed by chlamydia and trichomonas while male service members were most likely to have an etiologic diagnosis of chlamydia followed by herpes simplex virus (HSV) and gonorrhea.

**Table 4:** Case-rates (per 1,000 person-years) for individual STIs among women and men.

STI	Cases—women	Rate (95% CI)—women	Cases—men	Rate (95% CI)—men
Chlamydia	1,688	3.49 (3.33, 3.66)	907	0.69 (0.65,0.74)
Gonorrhea	513	1.06 (0.97, 1.16)	551	0.42 (0.39, 0.46)
HIV	19	0.04 (0.02, 0.06)	97	0.07 (0.06, 0.09)
HSV	1,127	2.33 (2.20, 2.47)	564	0.43 (0.40, 0.47)
HPV	2,547	5.27 (5.07, 5.48)	191	0.15 (0.13, 0.17)
Syphilis	69	0.14 (0.11, 0.18)	190	0.15 (0.13, 0.17)
Trichomonas	784	1.62 (1.51, 1.74)	93	0.07 (0.06, 0.09)

In multivariate analyses, factors associated with both etiologic and syndromic STIs among women included African American race, younger age and fewer years of education (Table 5). A longer period of military service was associated with increased likelihood of syndromic or pathogen-specific STIs among both men and women, while history of deployment was independently associated with increased likelihood of syndromic STI diagnosis in both women (OR 1.33 95% CI [1.24–1.42]) and men (OR 1.11 95% CI [1.00–1.18]). Men with greater time in the military were more likely to receive an STI diagnosis. Finally, racial patterns were differentially associated with syndromic and pathogen-specific STIs, as African American women were more likely to receive pathogen-specific diagnoses than syndromic diagnoses, in contrast with men, among whom syndromic diagnoses were more common.

**Table 5:** Multivariate analysis of factors associated with STI diagnosis during military service.

	Syndromic odds—women	Etiologic Odds—women	Syndromic odds -men	Etiologic odds—men
**Age**	0.68 (0.6–0.92)	0.88 (0.8–0.97)	0.82 (0.69–0.98)	1.02 (0.85–1.23)
**Race-Black**	1.78 (1.65–1.91)	2.28 (2.14–2.43)	3.36 (3.05–3.69)	0.96 (0.83–1.1)
**Race-Other**	1.22 (1.1–1.36)	1.02 (0.93–1.12)	1.1 (0.92–1.31)	0.81 (0.66–0.99)
**Rank-Officer**	0.84 (0.67–1.05)	0.75 (0.63–0.9)	0.75 (0.53–1.06)	1.06 (0.74–1.53)
**Deployed**	1.33 (1.24–1.42)	1.03 (0.97–1.1)	1.11 (1.0–1.22)	0.96 (0.86–1.08)
**Single**	1.05 (0.94–1.17)	0.77 (0.71–0.84)	1.34 (1.12–1.61)	0.71 (0.61–0.84)
**Service Years**	1.14 (1.13–1.15)	1.2 (1.19–1.21)	1.13 (1.12–1.14)	1.15 (1.14–1.16)

## Discussion

We found a high career prevalence of STI diagnoses among military men and women, consistent with prior studies of STIs in the military. Interestingly, the overall STI prevalence tended to increase throughout an individual’s length of military service. While the majority of STI diagnoses were syndromic, the percentage of women experiencing an STI was especially high, with nearly one-third receiving a pathogen-specific diagnosis at some point in their careers. Lastly, as with prior studies of military populations, we found that increased prevalence of STIs was associated with younger age [3,5,10] and African-American race [3,5,11–13].

The sustained increase over time in individual STI diagnoses was a surprising finding. As expected, STI prevalence increased most dramatically earlier in military service, as the cohort is younger and most likely to receive a new diagnosis. As the number of service-years increases, the proportion of individuals with an STI diagnosis remained constant and decreased after approximately six years of service, likely reflecting the retirement of enlisted personnel who tend to have higher STI prevalence. However, after eight years of service, a significant rise in the proportion of individuals with an STI diagnosis was observed, suggesting ongoing transmission among individuals previously believed to be at lower risk for STI acquisition. Military personnel with longer time in service tend to be older, more likely to be married, and have higher education levels, which are all protective factors in most other studies of STIs in the military. Thus, our findings suggest that there continues to exist an ongoing risk for STI acquisition during military service which may be higher than previously appreciated.

The disparity in STI prevalence between women and men likely reflects, at least in part, improved case identification due to screening practices among women; the magnitude of the difference suggests that STIs among military men are not being adequately identified and treated. While prior studies involving random samples of women and men have found higher incidence and prevalence of STIs among women, typically the differences are two- to three-fold, as opposed to the significantly larger differences that we report [3,14–18]. To reduce the prevalence as well as transmission of STIs in the military, these findings suggest a significant benefit to enhancing male-directed screening and diagnostic interventions. Additional measures, such as expedited partner therapy (EPT), which currently is not routinely offered in the military health system, might also provide benefit as has been shown in other high risk settings.

Military STI screening policies may also influence the comparability of data with those from civilian populations. All service women receive well-women exams on an annual basis, and both men and women are screened for HIV infection before deployment. In addition, service members receive HIV screening every other year which is not standard practice for the civilian population as a whole. Finally, military personnel undergo initial hepatitis screening upon entry into service, a practice which is not routinely performed among civilians. Beginning in 2001, the Armed Forces Epidemiological Board (now the Defense Health Board) recommended chlamydia screening for for all female recruits within one year of accession, as well as for all female service members at the time of routine pap smear up to age 25. The Navy and Marine Corps were the first military services to implement universal screening of recruits in the mid-1990s and the Air Force and Coast Guard followed suit in the mid-2000s. By 2008, all females in these services were being screened during recruit training using nucleic acid amplification tests (NAATs) while the Army continued screening only at annual exams. Our data suggests that service members are more likely to be screened for STIs than their civilian counterparts with only 44% of civilian women less than age 25 being screened in accordance to guidelines per Healthcare Effectiveness Data and Information (HEDIS) data from 2008 [19]. The disparity between genders is likely explained by screening among women that is not performed among males.

Among women, infection with human papilloma virus (HPV) was the most common pathogen-specific diagnosis, which is consistent with previous analysis of the military population during this period [20]. While the Food and Drug Administration (FDA) approved Gardasil in 2006 and Cervarix vaccines in 2009, the vaccines are not required on entry into military service, and as a result, uptake remains low. Hala-Maktabi et al found that between 2006–11, only 24% of women eligible for HPV vaccination initiated the series [21]. A study performed at Womack Army Medical Center found that only 15% of women received the vaccine between 2006–2009, and of these, only 37% completed the series [20]. At the Naval Medical Center, San Diego (NMCSD), Gunther et al found completion rates of only 32% of women who initiated the vaccine series, and completion rates were lowest for active-duty females (16%) and males (3%) [22]. Last, LaRocque and Berry-Caban found a similar vaccination rate of 15% among Army Soldiers at Fort Bragg North Carolina, from 2007–2010 [20]. The benefits of HPV vaccination are well-known, and even with suboptimal coverage, decreased rates of genital warts have been observed in the military since the introduction of HP vaccine [21]. Our paper again demonstrates the impact of HPV on sexual health and highlights the need for vaccination.

This study also found high rates of chlamydia among both male and female service members, whereas the overall prevalence for men and female service members was 1% and 5%, respectively. This prevalence is consistent with previous studies of the military population. Based on data from the National Health and Nutrition Examination Survey (NHANES), the US Centers for Disease Control and Prevention (CDC) estimated the prevalence of chlamydia among US males and females aged 14–39 to be 1.4% and 2.0%, respectively [19]. Similarly, Hakre et al noted that from 2005 to 2010, the Fort Bragg and surrounding Cumberland County, North Carolina, had STI rates that were twice that of the state as a whole [3]. Sena et al examined the Fort Bragg population from 1985 to 1996 and noted that while the adjusted incidence of gonorrhea infections declined to below the adjusted North Carolina rates, the rates of chlamydia infections were 3-to-6 times higher than the state and national levels [18]. In these studies, higher chlamydia prevalence may partially reflect increased compliance with US Preventive Services Task Force (USPSTF) guidelines through military screening; however, a higher baseline prevalence does seem likely based on the magnitude of the disparity.

Our study presents additional information on the relationship between deployment and STI incidence and prevalence. Interestingly, STI rates were higher among individuals without a history of deployment, though individuals without a history of deployment were less likely to be diagnosed with an STI than those with a deployment history. The reasons for this apparent discrepancy are unclear, and may reflect more frequent screening in non-deployed settings (i.e. higher rates), along with longer time in military for individuals with a deployment history, coinciding with a higher likelihood of STI diagnosis, as we have established. The effect of deployment on STI diagnosis has been seen in other studies; Hakre et al found that deployment was associated with lower incidents of STI diagnosis and recurrence [3]. Elsewhere, Aldous et al found that between 2005–09, STI prevalence increased among military personnel in Iraq and Afghanistan, likely reflecting a maturation of theater [14]. In our own survey, the combat environment may have affected documentation, screening, and treatment which was not captured in the medical record as well as increasing risk for STI. Further research is necessary to better define the relationship between deployment and STI diagnosis.

There are several limitations to this study. This study is retrospective, and the use of clinical data (i.e. diagnosis codes) as opposed to laboratory records for data collection may overestimate the prevalence of STIs, and indeed, we noted a high proportion of syndromic relative to pathogen-specific diagnoses. Additionally, the military’s screening policy and compliance were not constant during this study which may have affected the incidence of STI diagnoses in this study. Individuals may also be diagnosed with STIs outside the military system (e.g. public health clinics), which would reduce the observed versus actual prevalence. Still, our reported prevalence was high, indicating that most likely this did not occur to a significant extent. Finally, healthcare encounter data are also subject to errors in documentation and were partly collected during transitions between paper to electronic medical record (EMR); increased utilization of the EMR may have led to increases in case reporting during the later years of this study. The observed trend of increasing STI, however, was seen even in later years, when EMR use was consistent.

The strengths of this study include its large sample size, which would overcome many of these limitations and smooth potential inaccuracies. In addition, our analysis by calendar quarter allowed us to examine re-infection rates that may be missed by other research methods. Demographics of such a large sample naturally mirror those of the overall military, mitigating any sampling bias. In fact, the large population allowed for significant analysis to occur on less common STIs which have not been thoroughly analyzed in this population. While a number of cross-sectional studies have shown high prevalence of STIs, our analysis represents the first effort to follow a specific, large cohort to determine STI risk over time.

## Conclusions

We found a high burden of STIs in all branches of the military, and increasing years of service were associated with continued increases in new STI diagnoses. This study reinforces the need for ongoing STI-related screening and educational efforts throughout the military career of all service members.
